# Repetitive Pertussis Toxin Promotes Development of Regulatory T Cells and Prevents Central Nervous System Autoimmune Disease

**DOI:** 10.1371/journal.pone.0016009

**Published:** 2010-12-30

**Authors:** Martin S. Weber, Mahdia Benkhoucha, Klaus Lehmann-Horn, Deetje Hertzenberg, Johann Sellner, Marie-Laure Santiago-Raber, Michel Chofflon, Bernhard Hemmer, Scott S. Zamvil, Patrice H. Lalive

**Affiliations:** 1 Department of Neurology, Technische Universität München, Munich, Germany; 2 Department of Neurology, University of California San Francisco, San Francisco, California, United States of America; 3 Department of Neurosciences, Division of Neurology, Geneva University Hospital and University of Geneva, Faculty of Medicine, Geneva, Switzerland; 4 Department of Pathology and Immunology, Geneva University Hospital and University of Geneva, Faculty of Medicine, Geneva, Switzerland; 5 Department of Genetics and Laboratory Medicine, Geneva University Hospital, University of Geneva, Faculty of Medicine, Geneva, Switzerland; New York University, United States of America

## Abstract

Bacterial and viral infections have long been implicated in pathogenesis and progression of multiple sclerosis (MS). Incidence and severity of its animal model experimental autoimmune encephalomyelitis (EAE) can be enhanced by concomitant administration of pertussis toxin (PTx), the major virulence factor of *Bordetella pertussis*. Its adjuvant effect at the time of immunization with myelin antigen is attributed to an unspecific activation and facilitated migration of immune cells across the blood brain barrier into the central nervous system (CNS). In order to evaluate whether recurring exposure to bacterial antigen may have a differential effect on development of CNS autoimmunity, we repetitively administered PTx prior to immunization. Mice weekly injected with PTx were largely protected from subsequent EAE induction which was reflected by a decreased proliferation and pro-inflammatory differentiation of myelin-reactive T cells. Splenocytes isolated from EAE-resistant mice predominantly produced IL-10 upon re-stimulation with PTx, while non-specific immune responses were unchanged. Longitudinal analyses revealed that repetitive exposure of mice to PTx gradually elevated serum levels for TGF-β and IL-10 which was associated with an expansion of peripheral CD4^+^CD25^+^FoxP3^+^ regulatory T cells (Treg). Increased frequency of Treg persisted upon immunization and thereafter. Collectively, these data suggest a scenario in which repetitive PTx treatment protects mice from development of CNS autoimmune disease through upregulation of regulatory cytokines and expansion of CD4^+^CD25^+^FoxP3^+^ Treg. Besides its therapeutic implication, this finding suggests that encounter of the immune system with microbial products may not only be part of CNS autoimmune disease pathogenesis but also of its regulation.

## Introduction

Evidence suggests that in the pathogenesis of multiple sclerosis (MS), viral or bacterial agents may trigger or mislead activation of an immune system with the general potential to generate a self-reactive immune response [1], [2], [3]. Among viral candidates, association with development or progression of MS has been reported extensively for human herpes viruses (HHV), such as HHV-6 [4], [5], [6], [7] or Epstein Barr Virus (EBV, summarized in [8]). It remains to be determined whether this association is causative and whether one particular microorganism is specifically involved in MS pathogenesis. The bacterium *Bordetella pertussis* causes whopping cough in humans and produces pertussis toxin (PTx) as its main virulence factor. Like many common childhood infections, whopping cough acquired at young age is not significantly associated with later development of MS [9], whereas it is well supported that in patients with established MS systemic infections can trigger T cell activation which is associated with an elevated risk to develop a clinical relapse [10].

In the animal model of MS, experimental autoimmune encephalomyelitis (EAE), PTx is used to increase disease incidence and severity when administered simultaneously with the autoimmune challenge. The mechanism by which PTx administration facilitates EAE development is complex and not entirely understood. Structurally, PTx is composed of five proteins (S1, S2, S3, S4, and S5) and belongs to the A–B class of exotoxins [11]. The B subunit contains S2–S5 and binds to the surface of many eukaryotic cells. The A subunit S1, is subsequently released into the cytoplasm where it interferes with the inhibitory activity of Gi proteins unleashing intracellular signaling [12]. Pro-inflammatory activity of PTx is mainly attributed to an increased permeabilization of the otherwise cell-restrictive blood-brain barrier leading to an influx of immune cells into the CNS [13], [14]. This assumption may however not be conclusive as recent data suggest that PTx increases expression of cerebrovascular adhesion molecules [15], [16], proposing an alternative mechanism by which PTx may facilitate leukocyte migration into the CNS. PTx further promotes maturation and functional capacity of antigen presenting cells (APC) [17], increases production and release of pro-inflammatory cytokines such as IL-12 [18] and decreases secretion of anti-inflammatory IL-10 [19]. When used as an adjuvant for EAE induction, PTx reduces number and function of Treg [20], [21], while promoting development of encephalitogenic Th17 cells[22], [23]. Taken together, PTx may utilize multiple mechanisms to promote development of EAE.

Several primarily pro-inflammatory bacterial agents including PTx appear to also have protective properties when the immune system encounters them under certain circumstances. In this regard, pre-exposure of mice to Bordetella pertussis itself protected from subsequent EAE induction [24]. This effect could be attributed to the toxin produced, as genetically altered PTx failed to suppress CNS autoimmune disease [25]. Notwithstanding these initial observations, they left unclear how PTx facilitates EAE in one setting but may hinder its induction in another setting. In our study, we demonstrate that mice continuously exposed to PTx are indeed protected from active EAE induction which was associated with a markedly decreased proliferation and pro-inflammatory differentiation of myelin-reactive T cells. Most importantly, we report here that PTx treatment prior to disease induction elevated serum levels for TGF-β and IL-10 and promoted expansion and suppressive function of Treg providing an explanation on how repetitive exposure to PTX may prevent development of CNS autoimmune disease.

## Results

### Continuous PTx treatment is not immunosuppresive and does not induce tolerization

**Figure 1:**
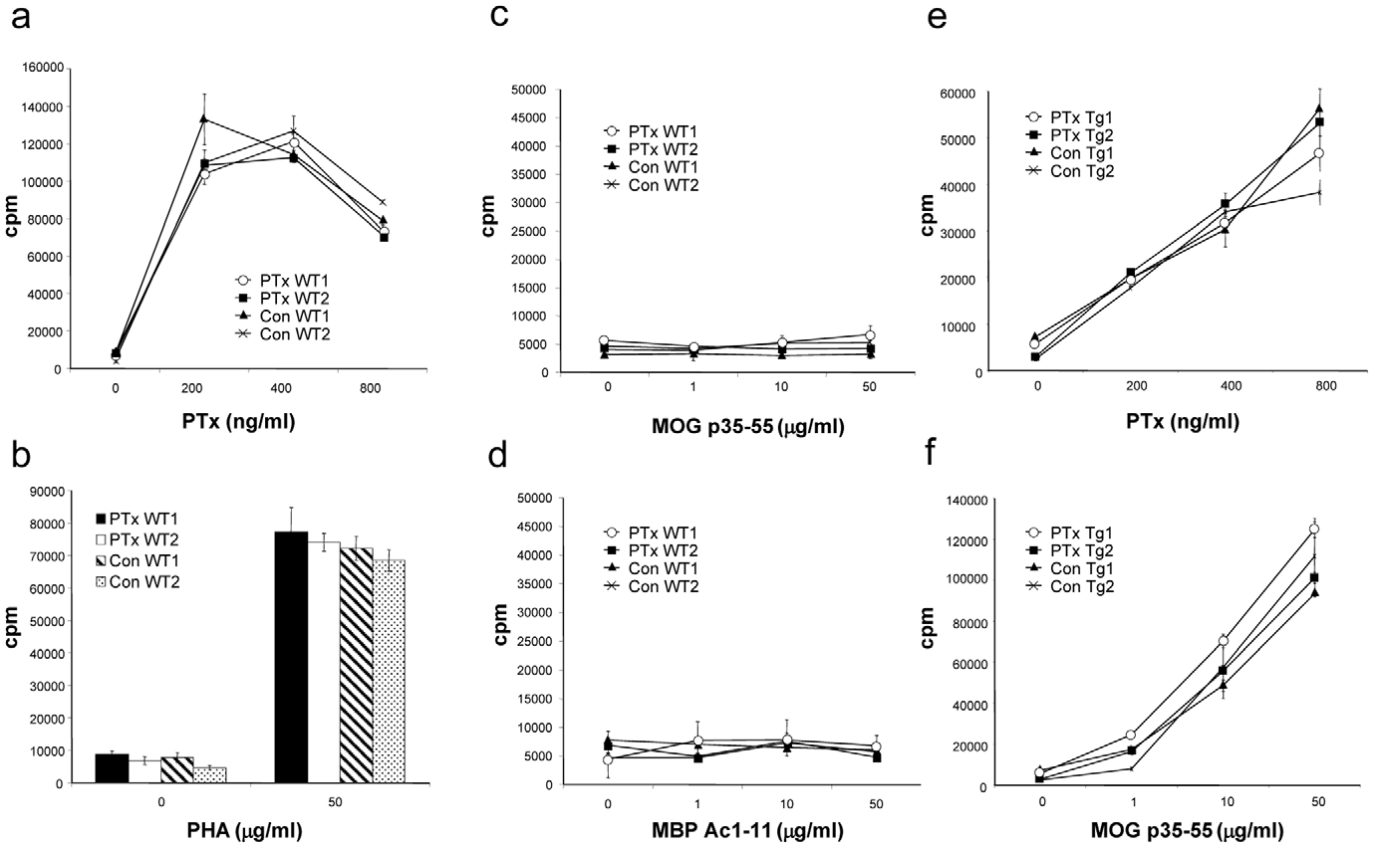
C57Bl/6 wild-type (12/group) (a–d) or MOG p35-55 TCR transgenic mice (6/group) (e, f) received weekly i.v. injections with 300 ng PTx in 200 ul of PBS or PBS alone for six months. Splenocytes from two representative mice per group were cultured in the presence of various concentrations of pertussis toxin (PTx) (**a**, **e**), phytohemagglutinin (PHA) (**b**), MOG p35-55 (**c**, **f**) or MBP Ac1-11 (**d**) (representative for two separate experiments).

First, we investigated whether continuous PTx pre-treatment may exert an unspecific immunosuppressive effect or may have tolerized mice for PTx, possibly hindering subsequent EAE induction using this particular adjuvant. Representative mice in both the PTx pre-treated, as well as in the control-treated group were sacrificed before EAE immunization and T cell responses to myelin antigens, PTx, and another mitogen, phytohaemagglutinin (PHA) were analysed (**Fig. 1a–d**). Proliferation of splenocytes in response to stimulation with PTx was indistinguishable in PTx pre-treated and control-treated mice (**Fig. 1a**). Similarly, no difference in proliferation was observed upon non-specific stimulation with PHA between PTx pre-treated and control-treated mice (**Fig. 1b**). Thus, continous PTx pre-treatment had not impaired its mitogenic potential and had not exerted an apparent tolerizing or immunosuppressive effect.

We next examined whether continuous PTx treatment may have generated myelin-specific T cell responses due to its presumed ability to increase permeabilization of the blood-brain-barrier. As shown in **figure 1c and d**, no peripheral proliferative response to myelin antigen was observed though, either in the group treated with PTx or in the control-treated group. To further address the question whether repetitively PTx-facilitated transmigration of myelin-specific T cells may be sufficient to induce CNS autoimmune disease, MOG p35-55 T cell receptor (TCR) transgenic mice [26] were injected with PTx weekly over six months. Like wild-type mice, none of these mice, which are highly susceptible to EAE, developed clinical signs of paralysis throughout the time of treatment (**Table S1**). Further, similar to wild-type mice, PTx treatment did not alter T cell responses to PTx (**Fig. 1e**) or MOG p35-55 (**Fig. 1f**) in MOG p35-55 TCR transgenic mice either.

### Repetitive PTx pre-treatment prevents development of clinical and histological EAE

**Figure 2:**
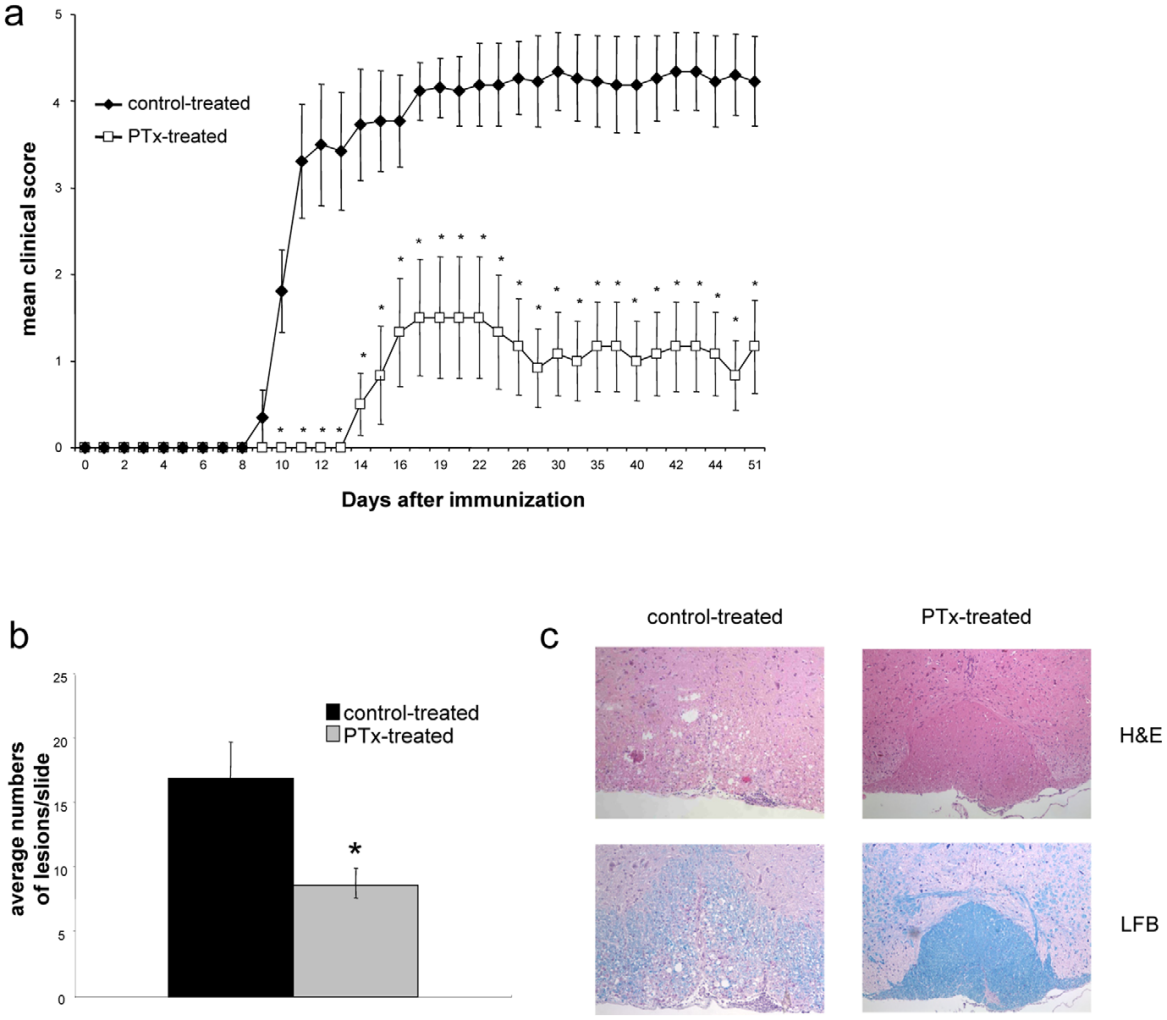
C57Bl/6 mice received weekly i.v. injections with 300 ng PTx in 200 ul of PBS or PBS alone. After six months of PTx injection, mice were immunized with MOG p35-55 in CFA and injected i.v. with 300 ng PTx in PBS immediately following the immunization and 48 h later. (**a**) Mice were followed for clinical signs of EAE (10 mice/group, mean severity is indicated as group average +/− SEM, * indicates p<0.05). (**b+c**) 50 days after EAE induction, CNS tissue from all mice was analysed for histological signs of EAE. Spinal cords were isolated and representative cervical, lumbal and thoracical sections were evaluated for inflammatory lesions. (**b**) Indicated is the average number of lesions per slide +/− SEM in each group (* indicates p<0.05). (**c**) Shown are representative slides stained with H&E (upper panels) or LFB (lower panels, magnification X100), (representative for two separate experiments).

Mice treated weekly with PTx were immunized with MOG p35-55 and evaluated for clinical signs of EAE. In comparison with the control-treated group, EAE was ameliorated in mice pre-treated with PTx (mean clinical score +/− SEM: 1.1 +/− 0.48 vs. 4.3 +/− 0.44; p = 0.0001). Further, onset of clinical symptoms was significantly delayed in the PTx pre-treated group (15 +/− 1 days) when compared to the control-treated group (10 +/− 7 days; p = 0.011) (**Fig. 2a**). Representative sections of the spinal cord and brain were compared between PTx and control-treated mice. The number of inflammatory lesions as well as the extent of inflammatory infiltration was lower in the PTx pre-treated group than in the control-treated group (mean number of inflammatory lesions per slide +/− SEM: 8,7 +/− 1.17 vs. 17 +/− 2.7; p<0.05) (**Fig. 2b**). Luxol fast blue staining indicated less or no demyelination in PTx pre-treated group whereas extensive areas of myelin loss were observed in the control-treated group (**Fig. 2c**).

### Repetitive PTx pre-treatment inhibits pro-inflammatory differentiation of myelin-specific T cells and promotes anti-inflammatory differentiation of PTx responsive cells

**Figure 3:**
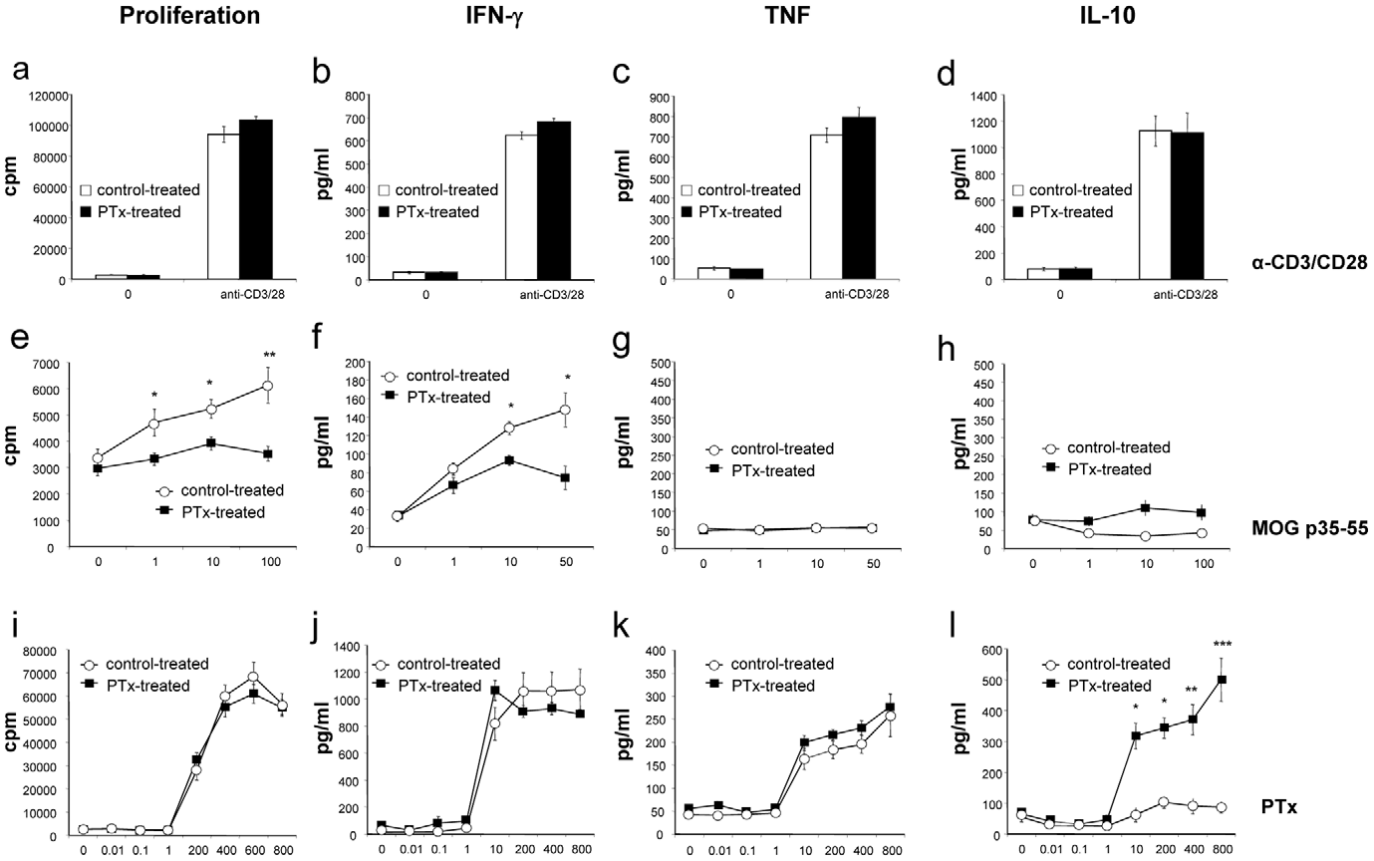
C57Bl/6 mice received weekly i.v. injections with 300 ng PTx in 200 µl of PBS or PBS alone. After six months, mice were immunized with MOG p35-55 in CFA and injected i.v. with 300 ng PTx in PBS immediately following the immunization and 48 h later. 50 days after EAE induction, isolated splenocytes from 4 (control-treated group) and 6 (PTx-treated group) representative mice were cultured with anti-CD3 (0,5 µg/ml) and anti-CD28 (1 µg/ml) (**a–d**), MOG p35-55 (**e–h**) or PTx (**i–l**) and evaluated for proliferation (**a**, **e**, **i**), secretion of IFN-γ (**b**, **f**, **j**), TNF (**c**, **g**, **k**) and IL-10 (**d**, **h**, **l**) (* indicates p<0.05, ** p<0.001, *** p<0.0001; representative for two separate experiments).

We next investigated T cell responses to myelin antigen after EAE induction comparing PTx- and control-treated mice. T cell proliferation and cytokine release in response to non-specific stimulation was similar between the PTx-treated and the control-treated group (**Fig. 3a–d**). In contrast, in the PTx-treated group, T cell proliferation was markedly reduced in response to MOG p35-55, the antigen used for EAE induction (**Fig. 3e**). In addition, production of the Th1 cytokine IFN-γ was decreased in the PTx pre-treated group upon re-stimulation with MOG p35-55 (**Fig. 3f**), but not with anti-CD3/CD28 (**Fig. 3b**) or PTx (**Fig. 3j**). Release of TNF, a pro-inflammatory cytokine mainly secreted by activated APC, was also analyzed. In contrast to IFN-γ, TNF production was not different between both groups neither upon stimulation with anti-CD3/CD28 (**Fig. 3c**), MOG 35-55 (**Fig. 3g**), or PTx (**Fig. 3k**). As we had observed that PTx pre-treatment protected mice from subsequent EAE induction, we investigated whether this pre-treatment had promoted development of anti-inflammatory cytokines with regulative capacity. Splenocytes from PTx-pre-treated mice released significant amounts of IL-10 specifically upon stimulation with PTx (**Fig. 3l**), but not following stimulation with antiCD3/CD28 (**Fig. 3d**) or re-stimulation with MOG p35-55 (**Fig. 3h**).

### Repetitive PTx pre-treatment promotes development of CD4^+^CD25^+^FoxP3^+^ Treg

**Figure 4:**
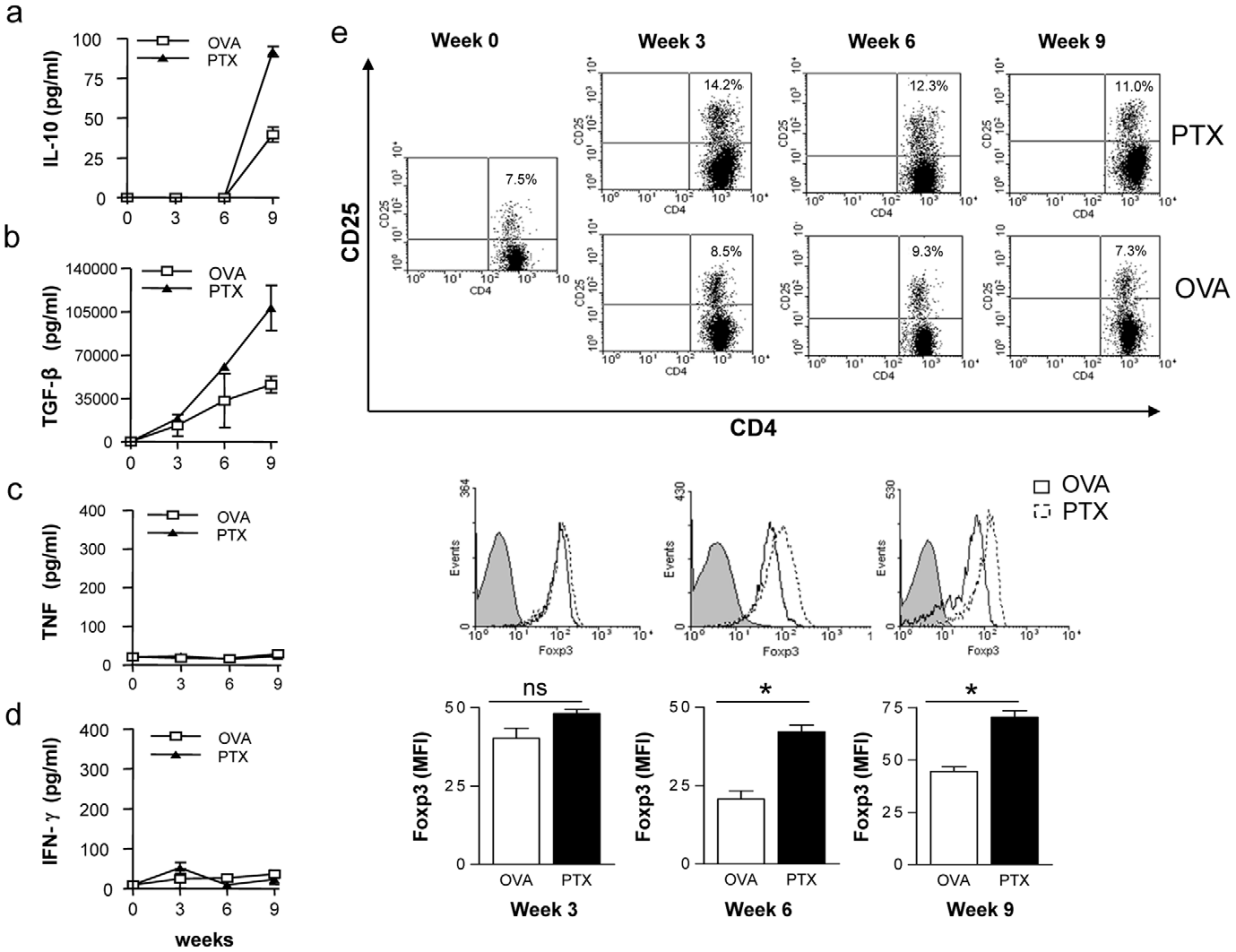
C57Bl/6 mice received weekly i.v. injections with 300 ng PTx or OVA 323–339 (control) in PBS. Serum cytokine IL-10 (**a**), TGF-β (**b**), TNF (**c**) and INF-γ (**d**) were determined at week 0-3-6-9 post PTx treatment. CD4^+^CD25^+^FoxP3^+^ Treg were analysed by flow cytometry at week 0-3-6-9 post PTx treatment (**e**; upper panels: indicated is the percentage of CD4^+^CD25^+^ within all CD4^+^ T cells; middle panels: FoxP3 expression of CD4^+^CD25^+^ cells; lower panels: mean FoxP3-FITC fluorescence intensity of CD4^+^CD25^+^ cells, * indicates p<0.05) (3 mice/group/time point; representative for three separate experiments).

Based upon this observation, we investigated next whether PTx treatment may enhance serum levels of anti-inflammatory cytokines. We thus injected mice weekly with PTx or the non-self antigen ovalbumin (OVA; 323–339). Serum cytokine levels were determined every 3 weeks after initiation of treatment. As shown in **figure 4a**, weekly injections with PTx elevated serum levels of IL-10 starting from week 9 after treatment initiation. Even more prominently, PTx injections raised serum levels of TGF-β (**Fig. 4b**) starting as early as 6 weeks after the first injection with PTx. In contrast, pro-inflammatory cytokines levels such as TNF (**Fig. 4c**) or IFN-γ (**Fig. 4d**) were not elevated.

**Figure 5:**
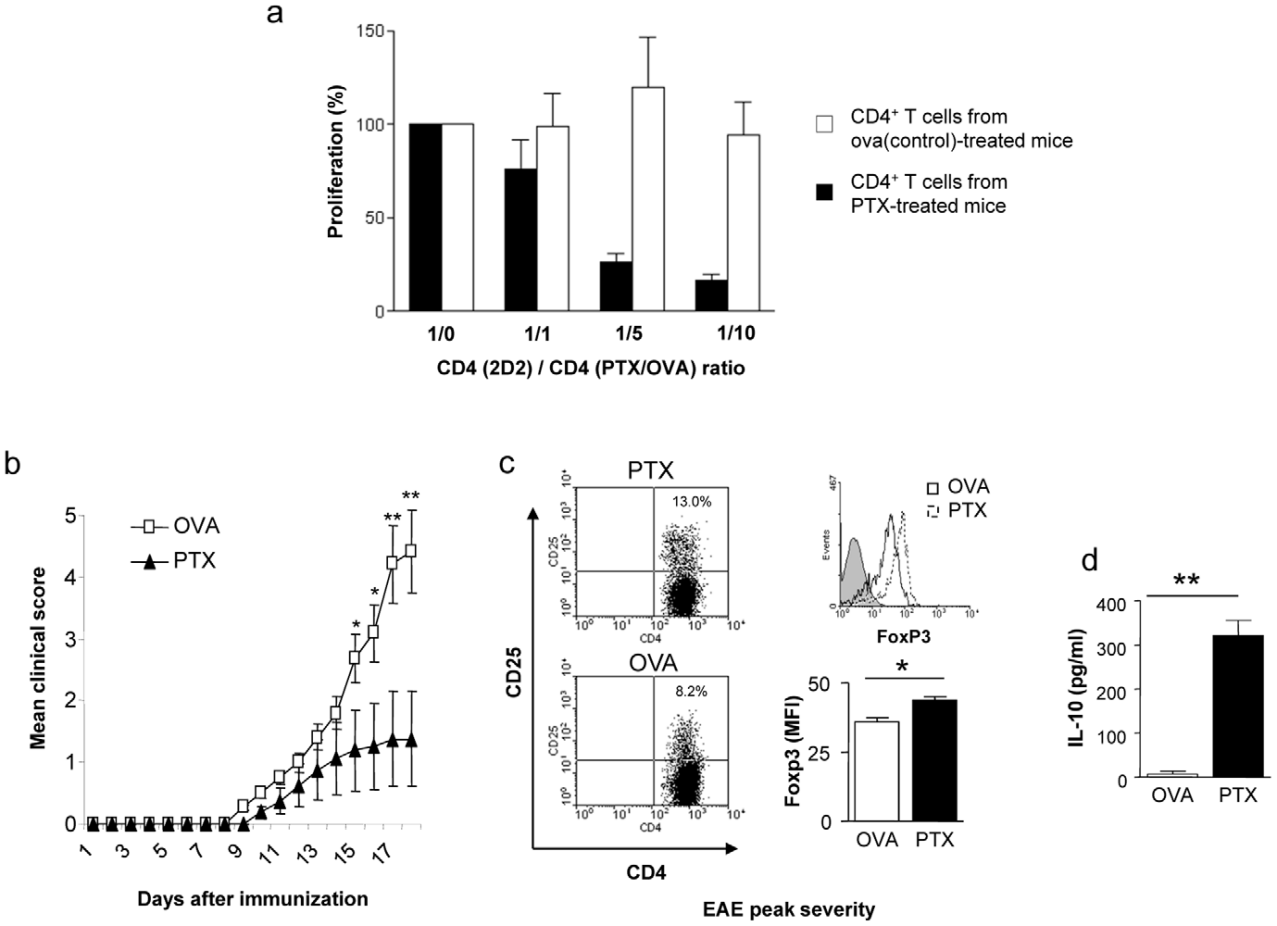
C57Bl/6 mice received weekly i.v. injections with 300 ng PTx or OVA 323–339 (control) in PBS. After 4 weeks, splenic CD4^+^ T cells were purified (3 mice/group) and co-cultured with 2×10^4^ dendritic cells and 4×10^4^ MOG p35-55 specific T cells from TCR transgenic (2D2) mice in the presence of 20 µg/ml MOG p35-55. Proliferation at the indicated ratio of MOG p35-55 specific CD4^+^ T cells (2D2)/CD4^+^ T cells from PTx-/ova-treated mice (PTx/ova) (1/1, 1/5, 1/10) is shown as percentage of the proliferative response without CD4^+^ T cells from PTx-/ova-treated mice (ratio 1/0) (**a**). After 10 weeks, EAE was induced with MOG p35-55 (8 mice/group). PTx pre-treatment significantly ameliorated disease severity (**b**). At the peak of clinical EAE severity, the frequency of CD4^+^CD25^+^FoxP3^+^ Treg and serum level of IL-10 was evaluated. Clinical benefit of PTx-pretreated mice was associated with expansion of FoxP3^+^ Treg cells (**c**; left panels: indicated is the percentage of CD4^+^CD25^+^ within all CD4^+^ T cells; right upper panel: FoxP3 expression of CD4^+^CD25^+^ cells; right lower panel: mean FoxP3-FITC fluorescence intensity of CD4^+^CD25^+^ cells) and elevated serum levels for IL-10 (**d**) (*indicates p<0.05, ** indicates p<0.001; representative for two separate experiments).

As TGF-β is centrally involved in development and maintenance of Treg [27], we analysed whether PTx pre-treatment was also associated with an increase in the frequency of CD4^+^CD25^+^FoxP3^+^ Treg. As shown in **figure 4e**, repetitive PTx treatment gradually elevated the frequency of splenic CD4^+^CD25^+^FoxP3^+^ Treg as well as the mean FoxP3 expression of CD4^+^CD25^+^ T cells, starting as early as 3 weeks after initiation of PTx treatment. In order to investigate whether these phenomenological changes would translate into an enhanced regulatory function within the CD4^+^ T cell compartment, we performed co-culture suppression assays. As displayed in **figure 5a**, CD4^+^ T cells from PTx-, but not from control-treated mice dose-dependently suppressed proliferation of myelin-specific T cells. Upon immunization (at week 10), PTx-treated mice -as expected- developed significantly less severe EAE compared to the OVA-treated control group **(** **Fig. 5b** **).** At the peak of disease severity, clinical benefit was associated with a sustained increase of Treg (**Fig. 5c**) and significantly elevated serum levels of IL-10 (**Fig. 5d**).

## Discussion

PTx is widely used as an adjuvant to induce EAE. Its co-administration with myelin antigen enhances incidence and severity of EAE and even renders otherwise resistant strains susceptible to EAE [13], [28]. This EAE-facilitating effect is not restricted to PTx, but has also been demonstrated for other bacterial antigens such as the superantigen staphylococcal enterotoxin B [29], [30], [31]. In our study, we first administered PTx weekly for six months in a dose used for EAE induction into mice susceptible to MOG-induced EAE. We hypothesized that through repetitive permeabilization of the blood-brain-barrier peripherally activated immune cells could infiltrate into the CNS where they may recognize myelin antigen. However, none of the mice continuously exposed to PTx developed myelin-specific T cell responses or showed any sign of paralysis throughout the course of PTx treatment. We also tested repetitive PTx treatment in MOG p35-55 TCR transgenic mice which contain a high frequency of myelin-recognizing T cells. 30% of these mice develop spontaneous optic neuritis, in a few cases combined with EAE symptoms [26]. However, while these mice were treated over a period of 6 months, repetitive PTx injections failed to increase EAE incidence in this strain as well.

In contrast, mice repetitively pre-treated with PTx were largely protected from subsequent EAE induction which is in accordance with earlier studies [24], [25]. Interestingly, in experimental autoimmune uveoretinitis (EAU) a comparable protective effect was observed with a single large dose of PTx [32], or when treatment begun after EAU was established [33]. Mice continuously exposed to PTx in our study exhibited markedly decreased proliferation and pro-inflammatory differentiation of myelin-reactive T cells upon immunization. So how could an agent with strongly pro-inflammatory, EAE-facilitating properties protect from the same disease when given repetitively prior to myelin antigen immunization? Several possibilities could account for this intuitively contradictive phenomenon. Most pro-inflammatory properties of PTx are frequently explained by its impairment of inhibitory G proteins. PTx was shown to enhance production of IL-12 and TNF facilitating development of Th1 responses [18]. More recent studies indicate that PTx administered as an adjuvant also promotes generation of IL-17-producing CD4^+^ T cells. In this regard, it has been demonstrated that PTx is required for efficient Th-1-, but even more so for Th-17 differentiation of myelin-specific T cells [23]. Another study could identify an enhanced release of IL-6 as the key factor for PTx-mediated pro-inflammatory Th-17 differentiation [22], which also occurred in models of other inflammatory conditions [34]. Presumably also through a transient upregulation of IL-6, PTx as an adjuvant to an immunogenic stimulus suppresses the frequency of CD4^+^CD25^+^FoxP3^+^ Treg [20], [21]. One possible explanation for the opposing effect of the repetitive application could have been that continuous activation may have triggered or facilitated T cell apoptosis [35]. Data indicate that activated T cells are particularly susceptible to apoptosis [36], [37], which could have decreased the frequency of self-reactive effector T cells before EAE induction. However, the unimpaired proliferation and pro-inflammatory differentiation of myelin-reactive T cells in repetitively PTx pre-treated MOG p35-55 TCR transgenic mice indicates that enhanced apoptosis of effector T cells is unlikely to account for the observed EAE resistance. Alternatively, continuous exposure to PTx could have induced T cell anergy [38], thereby specifically abolishing PTx's T cell activating property at the time of immunization. This assumption is fuelled by the fact that pre-treatment with Mycobacterium tuberculosis, which is also part of many active EAE induction protocols, similarly conferred protection against CNS autoimmune disease, whereas pretreatment with other bacterial components, e.g. from Escheriachia coli, Shigella or Staphylococcus aureus failed to do so [24]. However, at least in the case of Mycobacterium tuberculosis, it has been demonstrated that the protective effect could be attributed to a 12-kDa protein which failed to activate encephalitogenic T lymphocytes [39], suggesting that the domain conferring protection and the one facilitating EAE are not identical. The possibility of T cell anergy as the explanation for EAE resistance conferred by chronic PTx treatment appears further unlikely in the light of the unaltered immune cell response to PTx in both wild-type and MOG p35-55 TCR transgenic mice treated with PTx over several weeks.

In our study, PTx-mediated protection from CNS autoimmune disease was associated with an enhanced frequency and function of CD4^+^CD25^+^FoxP3^+^ Treg. Development of Treg was associated with elevated serum levels for TGF-β and IL-10, two cytokines centrally involved in development and maintenance of Treg [27], [40]. These regulatory cytokines are produced by a variety of immune cells, including T cells as well as various APC [41]. Although it remains to be determined which cell(s) released IL-10 and TGF-β in response to repetitive PTx exposure, our current study may provide a hint: Splenocytes isolated from PTx-treated mice secreted enhanced amounts of IL-10 specifically upon re-exposure to PTx, but not upon antigen non-specific T cell activation. These findings could suggest that IL-10 and TGF-β was rather than by T cells produced by APC, which may potently promote expansion of CD4^+^CD25^+^FoxP3^+^ Treg while presenting Ag to naïve T cells [42], [43].

In MS [44], as well as in other autoimmune conditions [45], frequency and function of Treg have been found to be impaired. Therapeutic restoration of Treg frequency to the levels of healthy control subject is associated with treatment benefit in MS [46]. Extensive investigations in mice have demonstrated that FoxP3^+^ Treg control development and severity of experimental CNS autoimmune disease (for review [47]): Adoptively transferred Treg significantly protected recipient mice from clinical EAE [48]; conversely, depletion of Treg has been shown to substantially increase EAE susceptibility as well as severity of its disease course [49], [50]. In our study, we have demonstrated that repetitive PTx treatment elevated frequency as well as mean FoxP3 expression of CD4^+^CD25^+^FoxP3^+^ T cells. PTx-expanded Treg efficiently suppressed proliferation of myelin-specific T cells. Taken together and in context with existing literature, these findings support the assumption that repetitive PTx administration may have protected mice from subsequent EAE induction trough prior expansion of Treg.

In summary, we identified that repetitive exposure to the bacterial antigen PTx gradually increased secretion of regulatory cytokines and expanded the population of Treg. This immunological alteration was associated with protection from CNS autoimmune disease. Besides its therapeutic implication, this finding indicates that in general, microbial products may not only be involved in the pathogenesis of CNS autoimmune disease but also in its regulation.

## Materials and Methods

### Ethics statement

All experiments carried out in this study were strictly performed in a manner to minimize suffering of laboratory mice. The individual protocols were approved by the respective committees at the University of California, San Francisco, USA (protocol approval number AN083156-01C), the Technische Universität München, Munich, Germany (protocol approval number 55.2-1-54-2531-67-09) and the University of Geneva (protocol ID number: 31.1.1005/3167/3).

#### Mice and EAE induction

C57BL/6 female mice, 5–8 weeks of age, were purchased from the Jackson Laboratory (Bar Harbor, MN). C57BL/6 MOG p35-55-specific TCR transgenic (2D2) mice [26] were kindly provided by Vijay K. Kuchroo (Harvard University) and Thomas Korn (Technische Universität München). Mouse myelin oligodendrocyte glycoprotein (MOG) peptide 35-55 (MEVGWYRSPFSRVVHLYRNGK) was synthesized and purified (>99%) by Auspep (Parkville, Australia). Age-matched mice were immunized subcutaneously (s.c.) with 25 µg MOG p35-55 in 0.1 ml of PBS emulsified in an equal volume of complete Freund's adjuvant (CFA) supplemented with 2 mg/ml of mycobacterium tuberculosis H37RA on day 0 (DIFCO Laboratories, Detroit, Michigan, USA). After immunization and 48 hrs later, mice received an intravenous (i.v.) injection of 300 ng of PTx in 0.2 ml of PBS [42], [51]. Individual animals were observed daily and clinical scores were assessed in a blinded fashion as follows: 0 =  no clinical disease, 1 =  loss of tail tone only, 2 =  mild monoparesis or paraparesis, 3 =  severe paraparesis, 4 =  paraplegia and/or quadraparesis, and 5 =  moribund or death.

#### Pertussis toxin treatment

PTx was purchased from List, Biological Laboratories Inc. (Campbell, USA). Age-matched mice received weekly i.v. injections with 300 ng PTx or OVA 323-339 (Abgent, Inc., San Diego, USA) in 0.2 ml of PBS or PBS alone.

#### Proliferation Assays

Primary proliferative responses were measured using splenocytes. 5×10^5^ spleen cells were cultured with antigen in 0.2 ml RPMI medium supplemented with 5×10^−5^ M 2-mercaptoethanol, 2 mM glutamine, 100 µg/ml penicillin, and 100 µg/ml streptomycin. Anti-mouse CD3/CD28 or PHA (BD Biosciences, Franklin Lakes, USA) were used as positive control. For co-culture assays, 2×10^4^ MACS (CD11c)-separated dendritic cells were used as APC together with 4×10^4^ MACS (CD4)-separated MOG p35-55-specific T cells from TCR transgenic (2D2) mice. In order to evaluate the suppressive capacity of Treg, increasing numbers of MACS (CD4)-separated T cells from PTx- or ova(control)-treated mice were added to the culture. After 72 hr, all proliferation assays were pulsed with 1 µCi [^3^H]-thymidine and harvested 16 hr later. Mean counts per minute (cpm) of [^3^H]-thymidine incorporation was calculated for triplicate cultures.

#### Cytokine analysis

Serum samples were obtained at the time-point indicated for cytokine analysis. Culture supernatants were collected at two time-points, 72-hr (TNF, IFN-γ, TGF-β), and 120-hr (IL-10). ELISA assays were performed using paired monoclonal antibodies specific for corresponding cytokines per manufacturer's recommendations (Pharmingen, San Diego, CA). The results for ELISA assays are expressed as an average of triplicate wells +/− SD. SOFTmax ELISA plate reader and software was used for data analysis (Molecular Devices Corporation, Sunnyvale, CA).

#### FACS analysis of CD4^+^CD25^+^ FoxP3^+^ Treg

Development of CD4^+^CD25^+^FoxP3^+^ Treg was evaluated using a FACS staining kit by eBiosience (San Diego, USA).

#### Histopathology

Brains and spinal cords of mice were fixed in 10% formalin. Sections were stained with Luxol fast blue-hematoxylin and eosin. Parenchymal inflammatory lesions were counted as described [52], [53].

#### Statistical analysis

Data are presented as mean ± SEM. For clinical scores significance between groups was examined using the Mann-Whitney *U* test. A value of *p*<0.05 was considered significant. All other statistical analysis was performed using a one-way multiple-range analysis of variance test (ANOVA) for multiple comparisons; individual *p* values are indicated.

## Supporting Information

Table S1 C57Bl/6 wild-type (WT) or MOG p35-55 T cell receptor (TCR) transgenic (Tg) mice received weekly i.v. injections with 300 ng PTx in 200 ul of PBS or PBS alone for six months. Mice were evaluated weekly for clinical signs of EAE.
(TIF)
