# Supplementary figures and images for: Repetitive Pertussis Toxin Promotes Development of Regulatory T Cells and Prevents Central Nervous System Autoimmune Disease
Source: PLoS One. 2010 Dec 30;5(12):e16009. doi: 10.1371/journal.pone.0016009 (PMC3012729; doi:10.1371/journal.pone.0016009)

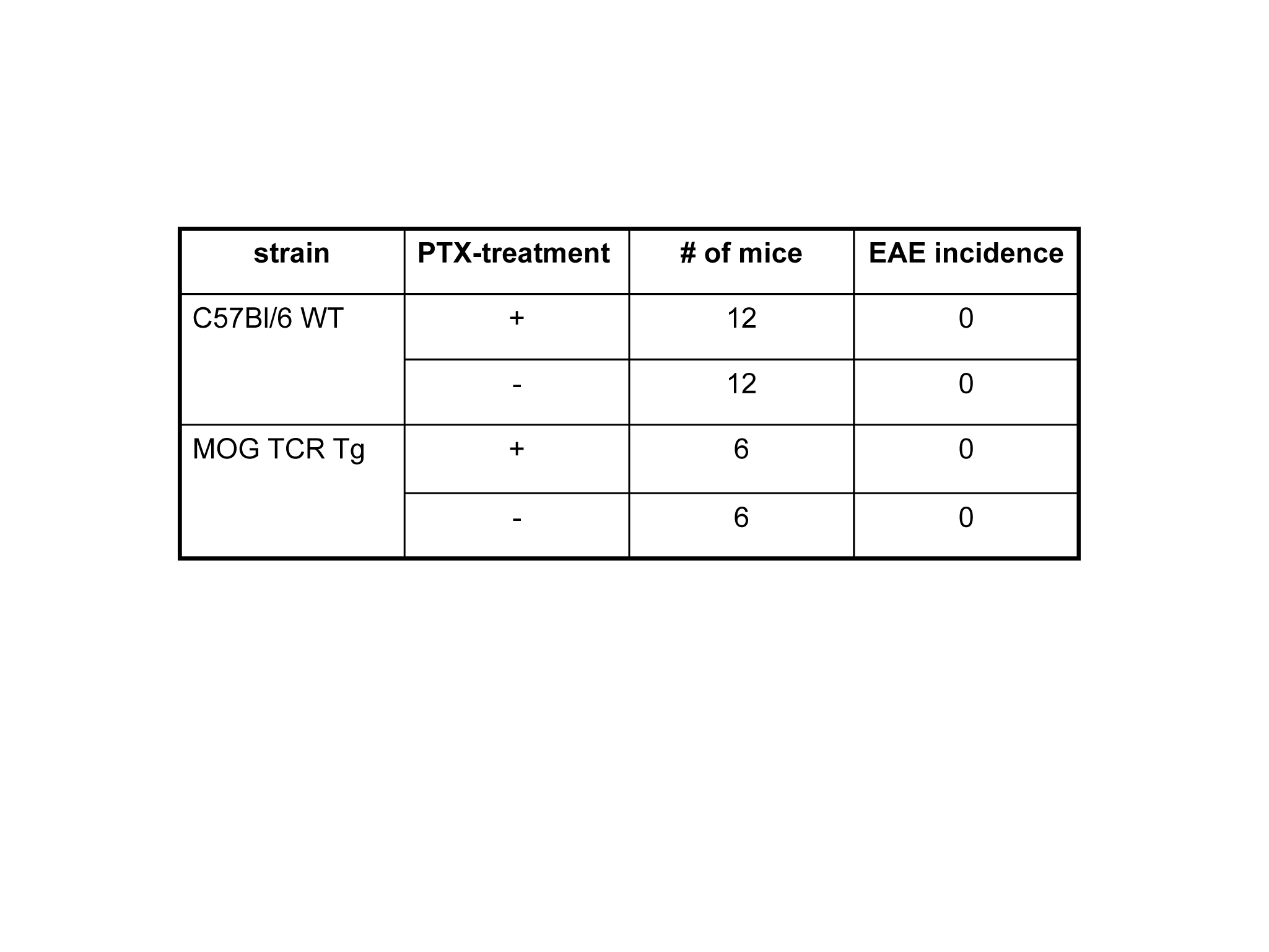

Supplement: Table S1 — C57Bl/6 wild-type (WT) or MOG p35-55 T cell receptor (TCR) transgenic (Tg) mice received weekly i.v. injections with 300 ng PTx in 200 ul of PBS or PBS alone for six months. Mice were evaluated weekly for clinical signs of EAE. (TIF) [file pone.0016009.s001.tif]
